# Discriminative Analysis of Schizophrenia Patients Using Topological Properties of Structural and Functional Brain Networks: A Multimodal Magnetic Resonance Imaging Study

**DOI:** 10.3389/fnins.2021.785595

**Published:** 2022-01-11

**Authors:** Jing Wang, Pengfei Ke, Jinyu Zang, Fengchun Wu, Kai Wu

**Affiliations:** ^1^School of Biomedical Engineering, Guangzhou Xinhua University, Guangzhou, China; ^2^School of Biomedical Sciences and Engineering, South China University of Technology, Guangzhou, China; ^3^The Affiliated Brain Hospital of Guangzhou Medical University, Guangzhou, China; ^4^Guangdong Engineering Technology Research Center for Translational Medicine of Mental Disorders, Guangzhou, China; ^5^Guangdong Engineering Technology Research Center for Diagnosis and Rehabilitation of Dementia, Guangzhou, China; ^6^National Engineering Research Center for Tissue Restoration and Reconstruction, South China University of Technology, Guangzhou, China; ^7^Key Laboratory of Biomedical Engineering of Guangdong Province, South China University of Technology, Guangzhou, China; ^8^Institute for Healthcare Artificial Intelligence Application, Guangdong Second Provincial General Hospital, Guangzhou, China; ^9^Department of Nuclear Medicine and Radiology, Institute of Development, Aging and Cancer, Tohoku University, Sendai, Japan

**Keywords:** schizophrenia, brain networks, discriminative analysis, machine learning, multimodal MRI

## Abstract

Interest in the application of machine learning (ML) techniques to multimodal magnetic resonance imaging (MRI) data for the diagnosis of schizophrenia (SZ) at the individual level is growing. However, a few studies have applied the features of structural and functional brain networks derived from multimodal MRI data to the discriminative analysis of SZ patients at different clinical stages. In this study, 205 normal controls (NCs), 61 first-episode drug-naive SZ (FESZ) patients, and 79 chronic SZ (CSZ) patients were recruited. We acquired their structural MRI, diffusion tensor imaging, and resting-state functional MRI data and constructed brain networks for each participant, including the gray matter network (GMN), white matter network (WMN), and functional brain network (FBN). We then calculated 3 nodal properties for each brain network, including degree centrality, nodal efficiency, and betweenness centrality. Two classifications (SZ vs. NC and FESZ vs. CSZ) were performed using five ML algorithms. We found that the SVM classifier with the input features of the combination of nodal properties of both the GMN and FBN achieved the best performance to discriminate SZ patients from NCs [accuracy, 81.2%; area under the receiver operating characteristic curve (AUC), 85.2%; *p < 0.05*]. Moreover, the SVM classifier with the input features of the combination of the nodal properties of both the GMN and WMN achieved the best performance to discriminate FESZ from CSZ patients (accuracy, 86.2%; AUC, 92.3%; *p < 0.05*). Furthermore, the brain areas in the subcortical/cerebellum network and the frontoparietal network showed significant importance in both classifications. Together, our findings provide new insights to understand the neuropathology of SZ and further highlight the potential advantages of multimodal network properties for identifying SZ patients at different clinical stages.

## Introduction

Schizophrenia (SZ) is a chronic psychiatric disease with hallucinations, delusions, and cognitive dysfunction (Lu et al., 2016). With the development of magnetic resonance imaging (MRI), the vast majority of studies have shown structural and functional brain abnormalities in SZ patients (Yamasue et al., 2004; Antonova et al., 2005; Kuroki, 2006; Pagsberg et al., 2007; Zhou et al., 2007). Several structural MRI studies have reported SZ patients show hippocampal volume reduction and bilateral thalamus volume reduction compared with normal controls (NCs) (Adriano et al., 2010, 2012), whereas the most reported functional alterations in SZ patients are located in thalamus, medial frontal gyrus, and superior temporal gyrus (Ren et al., 2013; Turner, 2013). Furthermore, previous studies have indicated that structural brain abnormalities are more widespread in chronic SZ (CSZ) patients than in first-episode drug-naive SZ (FESZ) patients, suggesting the potential impact of antipsychotic medication on structural brain abnormalities (Moncrieff and Leo, 2010; Torres et al., 2016). On the other hand, numerous functional MRI studies indicated that CSZ patients showed significant reductions in functional characteristics in brain regions involved in auditory, visual processing, and sensorimotor functions compared with FESZ patients (Wu et al., 2018). Therefore, it is critical to develop neuroimaging-based biomarkers for distinguishing the illness stages of SZ patients.

A number of studies based on the quantitative analysis of brain networks have reported that SZ patients show significantly decreased connectivity between a range of brain regions, particularly involving connections among the frontal lobe, temporal lobe, and insula compared with NCs (Bassett et al., 2008; Palaniyappan et al., 2015; Erdeniz et al., 2017). Previous studies of gray matter networks (GMNs) have shown that SZ patients exhibit reduced betweenness centrality (BC) in several regions and increased BC mainly in primary cortex and paralimbic cortex regions (Zhang et al., 2012). Numerous studies of white matter networks (WMNs) have demonstrated that a decreased clustering coefficient (Zhou et al., 2007; Collin et al., 2014), decreased global efficiency (van den Heuvel et al., 2013; Drakesmith et al., 2015; Griffa et al., 2015), and decreased node efficiency (NE) of the frontal lobe and limbic system were found in SZ patients (van den Heuvel et al., 2010; Wang et al., 2012; Sun et al., 2015). In addition, previous studies of functional brain networks (FBNs) indicated that the degree centrality (DC) in SZ patients was decreased in the bilateral putamen and increased in the left superior frontal gyrus (Chen et al., 2015). However, a few studies compared FESZ with CSZ patients based on the quantitative analysis of brain networks.

Recently, machine learning (ML) methods using neuroimaging data have been increasingly applied in the classification between SZ patients and normal controls (NCs), in which the classification accuracy varies from 0.65 to 0.95 (Arbabshirani et al., 2013; Talpalaru et al., 2019; Cao et al., 2020; Guo et al., 2020; Steardo et al., 2020). The majority of previous studies have mainly applied ML methods to a single neuroimaging modality, including structural MRI (sMRI) (Xiao et al., 2017; Oh et al., 2020), diffusion tensor imaging (DTI) (Ingalhalikar et al., 2010; Ardekani et al., 2011), resting-state functional MRI (rs-fMRI) (Koch et al., 2015; Skåtun et al., 2017; Cai et al., 2020), and electroencephalogram (EEG) (Ke et al., 2021). More recently, some studies have used multimodal MRI data to detect SZ at the level of the individual, and most measures are derived from multimodal MRI features, such as gray matter volume, regional homogeneity, amplitude of low-frequency fluctuation, and degree of centrality (Lu et al., 2018; Liang et al., 2019; Lei D. et al., 2020). Our previous study has applied multimodal MRI features to distinguish the illness stages of SZ patients based on ML methods and the findings have contributed to stage-specific biomarkers in diagnosis and interventions of SZ (Wu et al., 2018).

The objective of this study is to apply the features of both structural and functional brain networks derived from multimodal MRI data to the discriminative analysis of SZ patients at different clinical stages. We constructed three types of brain networks, including GMN, WMN, and FBN, for each participant derived from sMRI, DTI and rs-fMRI data. Three nodal properties of each brain networks were calculated, including BC, DC, and NE. The performance of two classifications (SZ vs. NC and FESZ vs. CSZ) was analyzed by using different combinations of nodal properties as the input features. Five ML algorithms were applied in both discriminative analyses, including the linear support vector machine (SVM) (Cortes, 1995), random forest algorithm (RF) (Breiman, 1996), logistic regression (LR) (Peng et al., 2002), linear discriminant analysis (LDA) (Fisher, 1936), and K-nearest neighbor classification (KNN) (Zhang, 2016).

## Materials and Methods

### Subjects

A total of 61 FESZ patients, 79 CSZ patients, and 205 NCs were recruited for this study. The SZ patients were diagnosed by trained and experienced clinical psychiatrists using a structured clinical interview according to Diagnostic and Statistical Manual of Mental Disorders: Fourth Edition, Text Revision (DSM-IV-TR) (Structured Clinical Interview for DSM Disorders [SCID]) criteria. The FESZ, CSZ, and NC groups were recruited from the Affiliated Brain Hospital of Guangzhou Medical University and the local community, respectively. All subjects were aged between 18 and 45 years, and their biological parents were Han Chinese. Before scanning, a clinical assessment was performed by psychiatrists using the Positive and Negative Syndrome Scale (PANSS) (Kay et al., 1987). The subjects obtained a consensus score for each item on all three subscales (positive symptoms, negative symptoms, and general psychopathology) that was based on a seven-point scale indicating the severity of the symptom (van Tol et al., 2014). The inclusion criteria for all SZ patients were as follows: (1) a total score of at least 60 for the three PANSS subscales and (2) at least 3 positive symptom items on the PANSS with a score of at least 4. Additionally, FESZ patients were recruited for the first time when they were seeking help due to psychotic symptoms and did not take any antipsychotics. All CSZ patients were taking antipsychotics, and the course of the disease was greater than 2 years.

The exclusion criteria for all subjects included (1) any other psychiatric Axis I disorder meeting DSM-IV criteria, including schizoaffective disorders, intellectual disability, major depressive disorder, bipolar disorder, delirium, dementia, memory disorder, and other cognitive disorders; (2) mental disorder due to substance dependence, a seriously unstable somatic disease, definite diabetes, thyroid diseases, hypertension, or heart disease; (3) narrow angle glaucoma; (4) a history of epilepsy, except for febrile convulsions; (5) alcohol dependence meeting DSM-IV-TR criteria (excluding nicotine dependence); (6) having received electroconvulsive therapy within the past 6 months; (7) a contraindication for MRI; (8) medical resource neuroleptic malignant syndrome or serious tardive dyskinesia; (9) a serious suicide attempt or an irritative state; (10) noncompliant drug administration or a lack of legal guardians; or (11) lactating, pregnant, planning to become pregnant. In addition, NCs were excluded if they had a first- or second-degree relative with a psychiatric Axis I disorder according to DSM-IV criteria. Before enrollment, all subjects or their legal guardians provided written informed consent. These studies were performed according to the Declaration of Helsinki and approved by the Ethics Committees of the Affiliated Brain Hospital of Guangzhou Medical University.

### Magnetic Resonance Imaging Data Acquisition

MRI images were acquired using a Philips 3T MR system (Philips, Achieva, Netherlands) located at the Affiliated Brain Hospital of Guangzhou Medical University. The participants were instructed to keep their eyes closed, to relax but not fall asleep, and to move as little as possible. The sMRI data were obtained using a sagittal three-dimensional gradient-echo T1-weighted sequence (256 × 256 × 188 matrix with 1 mm × 1 mm × 1 mm spatial resolution, repetition time (TR) = 8.2 ms, echo time (TE) = 3.8 ms, flip angle = 7°, field of view (FOV) = 256 mm × 256 mm). The DTI data were acquired using a single-shot echo-planar imaging-based sequence with the following parameters: slice thickness is 3 mm, no gap, 50 axial slices, TR = 6,000 ms; TE = 70 ms; flip angle = 90°; FOV = 256 mm × 256 mm; spatial resolution = 2 mm × 2 mm × 3 mm; 33 nonlinear diffusion weighting directions with b = 1000 s/mm^2^ and one image without diffusion weighting (b = 0 s/mm^2^). The rs-fMRI data were collected using an echo-planar imaging (EPI) sequence (64 × 64 × 36 matrix with 3.44 mm × 3.44 mm × 4 mm spatial resolution, TR = 2,000 ms, TE = 30 ms, flip angle = 90°, FOV = 220 mm × 220 mm).

### Image Processing

All T1-weighted MRI data processing was performed using the SPM8 software package^1^ (Institute of Neurology, University College London, United Kingdom). First, each T1-weighted MRI was segmented into three tissue maps, including gray matter (GM), white matter (WM), and cerebrospinal fluid (CSF), using the new segmentation algorithm from SPM8. Second, a customized, population-specific template was created from the segmented tissue maps using the DARTEL template-creation tool. Third, all GM maps were warped to the custom template space using its corresponding smooth, reversible deformation parameters. A modulation was applied by locally multiplying tissue values by the Jacobian determinants derived from the special normalization step (Good et al., 2001). Spatial smoothing was not performed to avoid inducing artifactual signal overlap among spatially adjacent regions (Kong et al., 2021).

Preprocessing of the DTI dataset was implemented using PANDA^2^, which is a pipeline toolbox for diffusion MRI analysis (Cui et al., 2013). The procedure mainly included skull stripping, simple motion and eddy current correction, and diffusion tensor/parameter calculation. Eddy current was an important factor for image deformation, and affine transformation was used to register the DTI image to the T1 image, which can effectively reduce the influence of head movement and eddy current.

Rs-fMRI data were preprocessed using SPM8 (see text footnote 1) Institute of Neurology, University College London, United Kingdom) and DPABI (Yan et al., 2016). First, the first 10 volumes of each functional time series were discarded because the initial signal was unstable. Second, the remaining volumes were corrected for different signal acquisition times and realigned to the first volume to correct for head motion. Then, the nuisance signals (Rigid-body 6 motion parameters, the white matter signal, and the cerebrospinal fluid signal) were regressed out from the data. Subsequently, all functional volumes were normalized using EPI templates and resampled to 3-mm isotropic voxels. The resampled data were bandpass (0.01–0.08 Hz) filtered to reduce low-frequency drift and high-frequency physiological noise and spatially smoothed with a 4-mm FWHM Gaussian kernel. All participants in the study did not have excessive head motion (<2 mm or 2° during realignment and <2 mm for mean motion during framewise assessment).

### Brain Network Construction

The single-subject GMN for T1-weighted MRI data was constructed based on the GM volume images. A GMN here included a collection of nodes and edges interconnecting the nodes. Here, the nodes represent brain regions, and edges represent interregional similarity in the distributions of regional GM volume. To define the network nodes, the GM volume image was parcellated into 268 regions of interest (ROIs) using a 268-node functional atlas (Shen et al., 2013). The GM volume value of each of the 268 ROIs was then calculated. We utilized a Kullback-Leibler (KL) divergence-based similarity (KLS) measure to estimate the network edges (Kong et al., 2014). Specifically, the probability density function of these 268 values was estimated using kernel density estimation (KDE) with bandwidths chosen automatically. The regional probability distribution function (PDF) and the KL divergence between each pair of 268 regions in their PDFs were then calculated. The network edges were defined as KLS with a consecutive sparsity threshold, S, ranging from 0.1 < S < 0.4 (interval = 0.01). All of the following network analyses were performed at each of the threshold levels in this range. For each of the network metrics, the estimated values under the range of 0.1-0.4 were integrated with area under curve (Wang et al., 2016).

The WMN for DTI data was also parcellated into 268 ROIs. According to deterministic white matter fiber bundle tractography, the fiber number (FN) connected in the ROIs was taken as the connection weight, and then the FN-weighted connection matrix was calculated. The parameters of fiber tractography were set: the turning angle between adjacent voxels was less than 45 degrees or fractional anisotropy was greater than 0.2 (Gong et al., 2009). The white matter connection was calculated using PANDA (Cui et al., 2013). The network edges were defined as the number of white matter fibers with the threshold FN > 2.

The FBN for fMRI data was constructed based on the main regional time series by averaging voxelwise time series data. Pearson’s correlation coefficient of the interregional time series was defined to measure the relation between network nodes. Then, each of the resulting correlation matrices was converted into a series of weighted networks with a sparsity threshold, S, ranging from 0.1 < S < 0.4 (interval = 0.01). All of the following network analyses were performed at each of the threshold levels in this range. For each of the network metrics, the estimated values under the range of 0.1-0.4 were integrated with area under curve (Liu et al., 2017).

The nodal properties of each region, including DC, NE, and BC, were then calculated using the GRETNA toolbox (Wang et al., 2015).

### Statistical Analysis

Between-group differences in age and years of education were analyzed by one-way analysis of variance (ANOVA) using SPSS 22.0 software. A *post hoc* analysis was performed using Scheffé’s method. A *χ^2^* test was performed to determine sex differences. Statistical significance was set at *p* < 0.05.

### Discriminative Analysis

In this study, we randomly split the set of participants into two groups, including a training dataset and an independent testing dataset, at a ratio of 4:1. In the training dataset, 10-fold cross-validation was performed. Each time, the ninefold data were used for training, and onefold data were used for validation. Each feature is normalized to between 0 and 1 on the training set, and the normalized parameters are applied to the validation set. Then, the performance of each resulting classifier was evaluated using the independent testing dataset. Using this method, we achieved unbiased estimates of every classifier.

**FIGURE 1:**
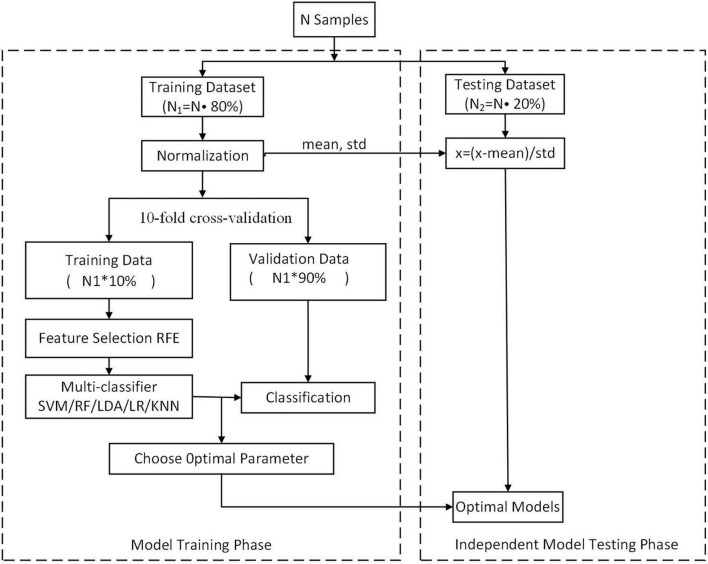
Flowchart of the ML classification method.

The performance of two classifications (SZ patients vs. NC and FESZ patients vs. CSZ) was analyzed using 5 ML algorithms, including SVM, RF, LR, LDA, and KNN. Moreover, we used the method of recursive feature elimination (RFE) (Lin et al., 2017) to iteratively remove redundant features while preserving discriminative features (Figure 1). The analyses for both classification tasks were performed by using the in-house software NEURO-LEARN^3^, which is a solution for collaborative pattern analysis of neuroimaging data (Lei B. et al., 2020).

**FIGURE 2:**
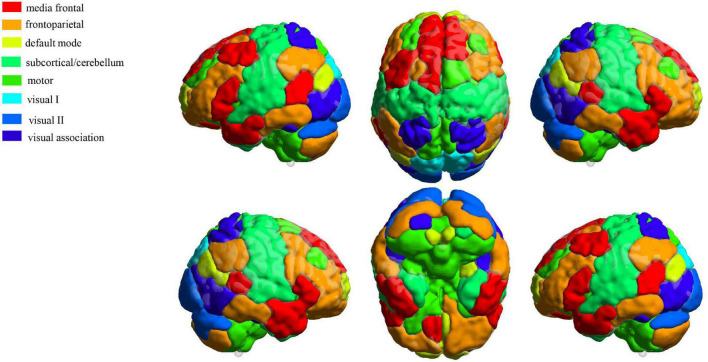
Definition of eight functional networks using a 268-node functional atlas. The figure was generated by using the toolbox BrainNet Viewer (http://www.nitrc.org/projects/bnv/).

From the results obtained by NEURO-LEARN, we obtained the weight of each input feature from the output of the classifier, and the absolute value of the feature weights can quantify the contribution of the features to the classifier. In this study, we calculated the feature contribution based on the results by the best classification performance. In addition, we divided 268 ROIs into eight subnetworks (Finn et al., 2015), including medial frontal, frontoparietal, default mode, subcortical/cerebellum, motor, visual I, visual II, and visual association (Figure 2). We selected the top 5% of combined features (BC, DC, and NE) ranked by the weights in the best classification to discuss subnetwork contributions.

To compare the performance of the 5 ML algorithms, we plotted the receiver operating characteristic (ROC) curves and calculated the area under the ROC curve (AUC). A permutation test was applied to explore whether the AUC obtained by the proposed model was significantly higher than the AUC of a random guess by randomly permuting the labels of the training data 1,000 times prior to the training step followed by the entire classification process. Based on probability distributions, it is possible to test the null hypothesis. The statistical significance was set at *p* < 0.05.

## Results

### Clinical and Demographic Characteristics

The clinical and demographic characteristics of all subjects are shown in Table 1. There were significant differences in age and years of education were noted between NCs and CSZ patients (*p < 0.05*); there were also significant differences in age and years of education between NCs and FESZ patients (*p < 0.05*). Moreover, there was no significant difference in the positive, negative, general, and total PANSS scores between FESZ and CSZ patients.

**TABLE 1:** Demographic and clinical characteristics.

	FESZ patients	CSZ patients	NC	Statistic value	*p* value
Sex (F:M)	41:20	54:25	110:95	χ^2^ = 3.53	0.03
Age (years)	32.08 ± 7.42	33.21 ± 8.37	32.52 ± 8.40	*F* = 5.39	<0.05^a^^,^^b^
Education (years)	10.39 ± 3.25	11.97 ± 3.22	12.84 ± 2.83	*F* = 21.33	<0.05^a^^,^^b^
PANSS-PScore	24.02 ± 4.50	22.47 ± 5.70	–	*T* = 1.74	0.083
PANSS-NScore	21.64 ± 7.70	23.22 ± 7.29	–	*T* = –1.24	0.218
PANSS-GScore	40.31 ± 8.85	39.54 ± 10.18	–	*T* = 0.47	0.641
PANSS-TScore	85.97 ± 17.49	85.23 ± 19.44	–	*T* = 0.23	0.816

*Values are represented as the mean ± standard deviation (SD). The comparisons of age and education among the three groups (FESZ, NC, and CSZ) were performed using a separate one-way ANOVA. Post hoc pairwise comparisons were then performed using a two-sample t-test. Statistical significance was set at p < 0.05. For the sex distribution among the three groups, the p value was obtained using the χ2 test.*

*^a^Post hoc paired comparisons showed significant group differences between CSZ vs. NC.*

*^b^Post hoc paired comparisons showed significant group differences between FESZ vs. NC.*

*CSZ, chronic schizophrenia; F, female; FESZ, first-episode drug-naive schizophrenia; GScore, general score; M, male; NC, normal control; NScore, negative syndrome score; PANSS, Positive and Negative Syndrome Scale; PScore, positive syndrome score; TScore, total syndrome score.*

### Classification Performance

Two classifications were performed to distinguish SZ patients from NCs as well as FESZ patients from CSZ patients. The network properties of GMN, WMN, and FBN were used as the input features of the five ML algorithms. The results indicated that the SVM achieved the best performance compared to the other four ML algorithms based on RFE in both classifications (Supplementary Tables 1, 2).

#### Results of Classification Between Schizophrenia Patients and Normal Controls

**FIGURE 3:**
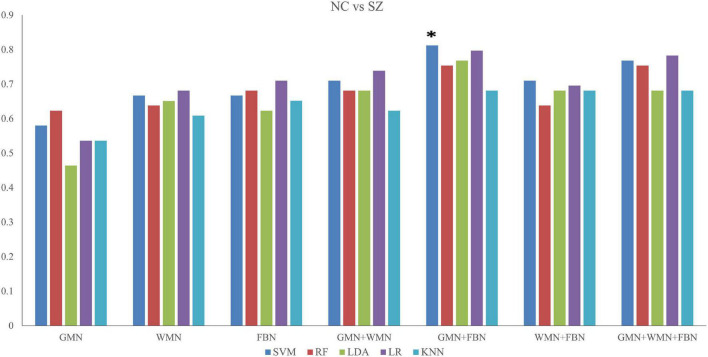
Accuracy values of the classifications between SZ patients and NCs based on five ML methods. *The star indicates the highest values of best performance of the classifications between SZ patients and NCs.

When discriminating SZ patients from NCs, the classifier of SVM with the input features of the properties of GMN and FBN achieved the highest performance with an accuracy of 81.2% and AUC of 85.2% (*p < 0.05*), as shown in Figure 3 and Supplementary Table 1.

**FIGURE 4:**
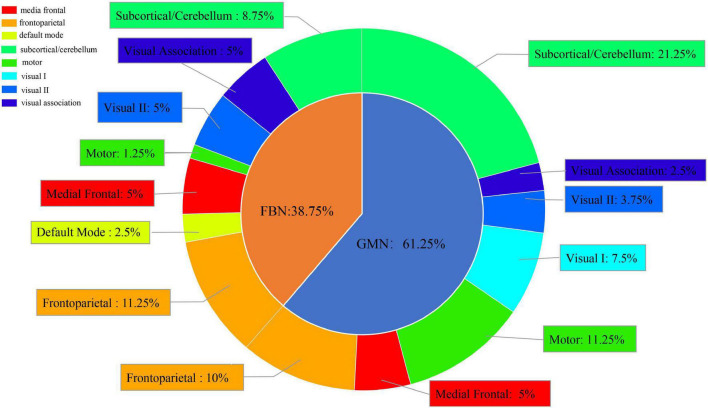
Subnetwork distribution of ROIs from the FBN and GMN in the best classification between SZ patients and NCs.

We chose the top 5% of features ranked by their weights in the best classification (Supplementary Table 3). Meanwhile, we divided 268 ROIs into eight subnetworks and calculated the frequency of each subnetwork in the top 5% of features from the GMN and FBN. We found that the ROIs from the GMN were distributed in the subcortical/cerebellum network, frontoparietal network, motor network, medial frontal network, visual I network, visual II network, and visual association network. The ROIs from the FBN were distributed in the subcortical/cerebellum network, frontoparietal network, medial frontal network, visual II network, visual association network, default mode network, and motor network (Figure 4).

Overall, the ROIs were mainly distributed in the subcortical/cerebellum network (30%) and frontoparietal network (21.25%), and the proportion of the features from the GMN was greater than the proportion of the features from the FBN in the classification between SZ patients and NCs.

#### Results of Classification Between First-Episode Drug-Naive SZ and Chronic SZ Patients

**FIGURE 5:**
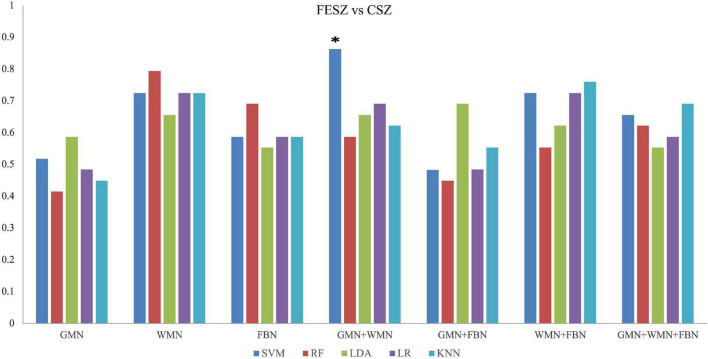
Accuracy values of the classifications between FESZ and CSZ patients based on five ML methods. *The star indicates the highest values of best performance of the classifications between FESZ and CSZ patients.

When discriminating FESZ from CSZ patients, the SVM classifier with the input features of the properties of GMN and WMN achieved the highest performance with an accuracy of 86.2% and AUC of 92.3% (*p < 0.05*), as shown in Figure 5 and Supplementary Table 2.

**FIGURE 6:**
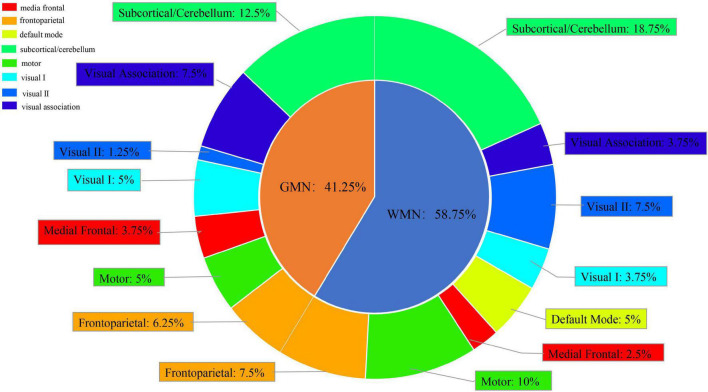
Subnetwork distribution of ROIs from the GMN and WMN in the best classification between FESZ and CSZ patients.

We chose the top 5% of features ranked by the weights based on the best classification combination (Supplementary Table 4). We found that the ROIs from the GMN were distributed in the subcortical/cerebellum network, frontoparietal network, visual association network, motor network, medial frontal network, visual I network, and visual II network. The ROIs from the WMN were distributed in the subcortical/cerebellum network, frontoparietal network, motor network, visual II network, default mode network, visual I network, visual association network, and medial frontal network (Figure 6).

Overall, the ROIs were mainly distributed in the subcortical/cerebellum network (31.25%), frontoparietal network (13.75%) and motor network (15%), and the proportion of the features from the WMN was greater than the proportion of the features from the GMN in the discrimination FESZ from CSZ patients.

## Discussion

In this study, for the first time, we discriminated SZ patients at different clinical stages using the complex network properties of the GMN, WMN, and FBN. Our main findings were as follows: (1) the SVM algorithm achieved the best performance compared to the other four ML algorithms in both classifications (SZ vs. NC and FESZ vs. CSZ); (2) the classifier with the input features of GMN and FBN achieved the highest performance in the classification between SZ patients and NCs; the classifier with the input properties of GMN and WMN achieved the highest performance in the classification between FESZ and CSZ patients; and (3) the features of ROIs in both subcortical/cerebellum and frontoparietal networks showed significant importance in both classifications.

The SVM algorithm, which determines a hyperplane that optimally distinguishes samples into two groups, has been widely used due to its reliable performance (Ding et al., 2015). In this study, our results indicated that the SVM achieved the best performance compared to the other four ML methods (including RF, LR, LDA, and KNN) in both classifications, which is consistent with previous studies of SZ (Chin et al., 2018; Rampisela and Rustam, 2018). A previous study discriminated SZ patients from NCs by analyzing multimodal brain imaging data with an SVM classifier and achieved good classification performance with a 91% accuracy and 100% prediction rate (Sui et al., 2014). Greenstein et al. (2012) applied the RF method to discriminate SZ patients from NCs using 74 anatomic brain MRI subregions and achieved 73.7% accuracy. Winterburn et al. (2019) performed a critical appraisal of the accuracy of ML methodologies used in SZ patient and NC classifications by comparing three ML methods (including SVM, LR, and LDA), and the highest accuracy achieved was 73.5% using SVM. Similarly, Li et al. (2015) evaluated the classification performance of four classification methods (including LDA, KNN, SVM, and Gaussian process classifier) on SZ diagnosis and obtained a maximum accuracy of 85.83% using the SVM. A recent study reviewed five traditional ML algorithms (including SVM, RF, KNN, gradient boosting machine and naive Bayes) frequently used for mental health and indicated that the advantage of SVM was working relatively accurately in general in practice (Cho et al., 2019). Our results confirmed the effectiveness of the SVM algorithm on SZ classification with the features of structural and functional brain networks derived from multimodal MRI data.

The classifier with the input features of the combination of the nodal properties of both GMN and FBN achieved the best performance (accuracy of 81.2%) compared to the classifiers using the input features of a single brain network when discriminating SZ from NC. Considerable evidence indicates that SZ is associated with structural as well as functional brain abnormalities (Oh et al., 2017; Li et al., 2020). A recent study revealed that the use of combined structural and functional measures allows the highest accuracy of classification to detect SZ (Lei D. et al., 2020). However, the input features of previous SZ classification studies were derived from structural and functional neuroimaging features (de Filippis et al., 2019; Shi et al., 2021). Our results based on brain network properties further highlight the potential advantages of multimodal features for distinguishing SZ patients from NCs.

Importantly, we also found that the classifier using the nodal properties of both the GMN and WMN achieved the best performance (accuracy of 86.2%) when discriminating FESZ from CSZ. The WMN from DTI data played a key role in predicting individuals with FESZ, which is consistent with previous studies (Lee et al., 2013; Samartzis et al., 2014). Friedman et al. (2008) indicated that fractional anisotropy of the white matter in the forceps minor or the genu of the corpus callosum showed striking differences between FESZ and CSZ patients based on DTI findings. White matter abnormalities in SZ as estimated by DTI appear to be present in the early stage of the disorder, most likely reflecting the developmental stage (Kyriakopoulos and Frangou, 2009; Liang et al., 2019). The main objective of our study is to analyze the importance of complex brain networks derived from multi-modal MRI data in the classification of SZ patients at different clinical stages. Our results showed that GMN and FBN played a more important role in discriminating SZ from NCs, whereas GMN and WMN played a more important role in discriminating FESZ from CSZ. Together, these findings may provide new insights into the neuropathology of SZ at various clinical stages.

In this study, our results demonstrated that the ROIs in the subcortical/cerebellar network and frontoparietal network showed significant importance in SZ classification. A large number of previous studies have indicated that SZ patients showed significantly altered brain connectivity involved in the frontoparietal network, cerebellum network and visual network (Wang et al., 2018; de Filippis et al., 2019). Interestingly, the frontoparietal network and the cerebellum network are among the most commonly implicated networks in SZ patients (Watanabe et al., 2014). The frontoparietal network, which has multiple important hubs in the prefrontal cortex, is involved in executive processing and cognitive control and has been shown to exhibit abnormal activation and connectivity in SZ patients (Minzenberg et al., 2009; Cole et al., 2013; Tu et al., 2013). The cerebellum network, which is featured in the influential ‘cognitive dysmetria’ hypothesis of SZ patients (Andreasen et al., 1998), has been found to be the most important network in the functional connectivity of SZ patients according to the extended maximal information coefficient (Su et al., 2013). Moreover, previous studies based on deep learning algorithms have discovered that cortical-striatal-cerebellar functional connectivity features exhibit great weights in the classification of SZ (Zeng et al., 2018; Qureshi et al., 2019). We also found that the ROIs in these two networks showed key importance in discriminating FESZ and CSZ patients. A meta-analysis of 25 articles based on cerebellar structural and functional abnormalities in FESZ patients hypothesized that the changes in both structural and functional aspects might reflect a common pathophysiology of the cerebellum in SZ patients (Ding et al., 2019). Lui et al. (2010) reported that 6 weeks of antipsychotic treatment increased brain synchronous activity in the frontal and parietal regions in FESZ patients, which might contribute to the importance of the frontoparietal network in discriminating FESZ and CSZ patients. In addition, the ROIs in the motor network also showed significant importance in discriminating FESZ patients from CSZ patients but not in SZ patients from NCs. It was reported that the functional connectivity in the sensory-motor network could reflect the hypothesized “neurotoxic effect” of FESZ patients (Zhang et al., 2019), demonstrating that the motor network deserves more attention in the search for neuroimaging markers for evaluating neural impairment in SZ.

Interestingly, we found that the BC of network topological features has more importance than DC and NE to the classifier for both contrast groups. The BC is a ratio of the number of all shortest paths between any two nodes in the network that travel through an index node, providing an indication of how topologically central the role of node is in overall network communication (van den Heuvel and Fornito, 2014). Previous brain network studies have indicated that BC in some brain regions is decreased (van den Heuvel and Hulshoff Pol, 2010; Zhang et al., 2012). Our study may further confirm the importance of the BC of network topological features in identifying SZ.

## Limitations of the Methodology

Possible study limitations should be considered. First, only three nodal features (BC, DC, and NC) were combined in the present study. Additional network measures, such as clustering coefficient, path length and global efficiency, can be used and may improve classification performance. Second, we compared the performance of five ML methods using only RFE as the dimensionality reduction algorithm. Previous studies of SZ patients have shown different classification performances using different dimensionality reduction algorithms (Li et al., 2015; Winterburn et al., 2019). Third, we have used different brain atlases in the discriminative analysis of SZ patients based on neuroimaging features and revealed that the 268-node functional atlas outperformed the other two brain atlases (Zang et al., 2021). However, it is necessary to compare the effects among different combinations of dimensionality reduction algorithms and brain atlases from the perspective of brain network properties in future studies. Fourth, the classification performance did not always benefit from more modalities in either classification. Previous studies have also shown that including more features does not necessarily yield positive effects due to feature redundancy. However, a recent study proposed a safe classifier that could address this issue to some extent (Hou et al., 2019). It is important to study whether safe classification would affect the discriminative results based on multimodal brain networks in future studies.

## Conclusion

We constructed GMNs, WMNs and FBNs from sMRI, DTI and fMRI data, respectively and then discriminated SZ patients at different clinical stages using different combinations of nodal properties based on five ML methods. Our results indicated the best performance of the SVM algorithm in SZ classifications and highlighted the potential advantages of multimodal network properties in identifying SZ patients at different stages. Furthermore, the ROIs in the subcortical/cerebellum network and frontoparietal network showed significant importance in both classifications (NC vs. SZ, FESZ vs. CSZ). Our findings may bring new insights into the understanding of the neuropathology of SZ from the perspective of network properties.

## Data Availability Statement

The raw data supporting the conclusions of this article will be made available by the authors without undue reservation.

## Ethics Statement

The studies involving human participants were reviewed and approved by Ethics Committees of the Affiliated Brain Hospital of Guangzhou Medical University. The patients/participants provided their written informed consent to participate in this study.

## Author Contributions

All authors contributed to data analysis, drafted and critically revised the manuscript, gave final approval of the version to be published, and agreed to be accountable for all aspects of the work.

## Conflict of Interest

The authors declare that the research was conducted in the absence of any commercial or financial relationships that could be construed as a potential conflict of interest.

## Publisher’s Note

All claims expressed in this article are solely those of the authors and do not necessarily represent those of their affiliated organizations, or those of the publisher, the editors and the reviewers. Any product that may be evaluated in this article, or claim that may be made by its manufacturer, is not guaranteed or endorsed by the publisher.

## Footnotes

1
http://www.fil.ion.ucl.ac.uk/spm

2
http://www.nitrc.org/projects/panda/

3
https://github.com/Raniac/NEURO-LEARN
