# Supplementary material for: Discriminative Analysis of Schizophrenia Patients Using Topological Properties of Structural and Functional Brain Networks: A Multimodal Magnetic Resonance Imaging Study
Source: Front Neurosci. 2022 Jan 11;15:785595. doi: 10.3389/fnins.2021.785595 (PMC8787107; doi:10.3389/fnins.2021.785595)
Supplement: Supplementary file 1 [file Table_1.DOCX]

Table 1 Classification performance based on five machine learning methods for

SZ-NC groups

| **Feature Selection method** | **Combi- nation** | **Classifier** | **Accuracy** | **Sensitivity** | **Specificity** | **AUC** | **P** |
| --- | --- | --- | --- | --- | --- | --- | --- |
| **Recursive Feature Elimination** | GMN | SVM | 0.580 | 0.500 | 0.634 | 0.639 | 0.0495 |
|  |  | RF | 0.623 | 0.214 | 0.902 | 0.646 | 0.0099 |
|  |  | LDA | 0.464 | 0.214 | 0.634 | 0.459 | 0.0198 |
|  |  | LR | 0.536 | 0.393 | 0.634 | 0.591 | 0.0396 |
|  |  | KNN | 0.536 | 0.357 | 0.659 | 0.537 | 0.0099 |
|  | WMN | SVM | 0.667 | 0.571 | 0.732 | 0.683 | 0.0099 |
|  |  | RF | 0.638 | 0.286 | 0.878 | 0.653 | 0.0099 |
|  |  | LDA | 0.651 | 0.607 | 0.683 | 0.629 | 0.0099 |
|  |  | LR | 0.681 | 0.571 | 0.756 | 0.691 | 0.0099 |
|  |  | KNN | 0.609 | 0.286 | 0.829 | 0.693 | 0.0099 |
|  | FBN | SVM | 0.667 | 0.679 | 0.659 | 0.716 | 0.0099 |
|  |  | RF | 0.681 | 0.429 | 0.854 | 0.742 | 0.0099 |
|  |  | LDA | 0.623 | 0.571 | 0.659 | 0.717 | 0.0099 |
|  |  | LR | 0.710 | 0.643 | 0.756 | 0.761 | 0.0099 |
|  |  | KNN | 0.652 | 0.571 | 0.707 | 0.707 | 0.0099 |
|  | GMN+WMN | SVM | 0.710 | 0.679 | 0.732 | 0.748 | 0.0099 |
|  |  | RF | 0.681 | 0.321 | 0.927 | 0.732 | 0.0099 |
|  |  | LDA | 0.681 | 0.500 | 0.805 | 0.744 | 0.0099 |
|  |  | LR | 0.739 | 0.714 | 0.756 | 0.758 | 0.0099 |
|  |  | KNN | 0.623 | 0.250 | 0.878 | 0.524 | 0.0099 |
|  | GMN+ FBN | **SVM** | **0.812** | **0.714** | **0.878** | **0.852** | **0.0099** |
|  |  | RF | 0.754 | 0.393 | 1.000 | 0.764 | 0.0099 |
|  |  | LDA | 0.768 | 0.643 | 0.854 | 0.838 | 0.0099 |
|  |  | LR | 0.797 | 0.714 | 0.854 | 0.861 | 0.0099 |
|  |  | KNN | 0.681 | 0.536 | 0.780 | 0.695 | 0.0099 |
|  | WMN+FBN | SVM | 0.710 | 0.679 | 0.732 | 0.797 | 0.0099 |
|  |  | RF | 0.638 | 0.429 | 0.780 | 0.701 | 0.0099 |
|  |  | LDA | 0.681 | 0.571 | 0.756 | 0.787 | 0.0099 |
|  |  | LR | 0.696 | 0.714 | 0.683 | 0.787 | 0.0099 |
|  |  | KNN | 0.681 | 0.536 | 0.780 | 0.760 | 0.0099 |
|  | GMN+WMN+FBN | SVM | 0.768 | 0.750 | 0.780 | 0.851 | 0.0099 |
|  |  | RF | 0.754 | 0.536 | 0.902 | 0.787 | 0.0099 |
|  |  | LDA | 0.681 | 0.536 | 0.780 | 0.776 | 0.0099 |
|  |  | LR | 0.783 | 0.750 | 0.805 | 0.865 | 0.0099 |
|  |  | KNN | 0.681 | 0.286 | 0.951 | 0.702 | 0.0099 |

Table 2 Classification performance based on five machine learning methods

for FESZ-CSZ groups

| Feature Selection method | Combi-nation | Classifier | Accuracy | Sensitivity | Specificity | AUC | P |
| --- | --- | --- | --- | --- | --- | --- | --- |
| Recursive Feature Elimination | GMN | SVM | 0.517 | 0.385 | 0.625 | 0.428 | 0.2178 |
|  |  | RF | 0.414 | 0 | 0.750 | 0.5 | 0.0495 |
|  |  | LDA | 0.586 | 0.615 | 0.563 | 0.623 | 0.0891 |
|  |  | LR | 0.483 | 0.385 | 0.563 | 0.418 | 0.1584 |
|  |  | KNN | 0.448 | 0.462 | 0.438 | 0.478 | 0.0099 |
|  | WMN | SVM | 0.724 | 0.615 | 0.813 | 0.813 | 0.0099 |
|  |  | RF | 0.793 | 0.692 | 0.875 | 0.865 | 0.0099 |
|  |  | LDA | 0.655 | 0.538 | 0.750 | 0.711 | 0.0099 |
|  |  | LR | 0.724 | 0.615 | 0.813 | 0.846 | 0.0099 |
|  |  | KNN | 0.724 | 0.538 | 0.875 | 0.803 | 0.0099 |
|  | FBN | SVM | 0.586 | 0.461 | 0.688 | 0.591 | 0.0693 |
|  |  | RF | 0.690 | 0.307 | 1 | 0.668 | 0.0495 |
|  |  | LDA | 0.552 | 0.538 | 0.563 | 0.550 | 0.0099 |
|  |  | LR | 0.586 | 0.462 | 0.688 | 0.505 | 0.1089 |
|  |  | KNN | 0.586 | 0.538 | 0.625 | 0.591 | 0.0198 |
|  | GMN+WMN | **SVM** | **0.862** | **0.846** | **0.875** | **0.923** | **0.0198** |
|  |  | RF | 0.586 | 0.231 | 0.875 | 0.822 | 0.0099 |
|  |  | LDA | 0.655 | 0.615 | 0.688 | 0.721 | 0.0099 |
|  |  | LR | 0.690 | 0.615 | 0.750 | 0.712 | 0.0099 |
|  |  | KNN | 0.621 | 0.385 | 0.813 | 0.736 | 0.0099 |
|  | GMN +FBN | SVM | 0.482 | 0.384 | 0.563 | 0.438 | 0.0099 |
|  |  | RF | 0.448 | 0.154 | 0.688 | 0.476 | 0.0297 |
|  |  | LDA | 0.690 | 0.538 | 0.813 | 0.514 | 0.0099 |
|  |  | LR | 0.483 | 0.385 | 0.563 | 0.423 | 0.0099 |
|  |  | KNN | 0.552 | 0.385 | 0.688 | 0.577 | 0.0099 |
|  | WMN+FBN | SVM | 0.724 | 0.615 | 0.813 | 0.769 | 0.0099 |
|  |  | RF | 0.552 | 0.538 | 0.563 | 0.688 | 0.0099 |
|  |  | LDA | 0.621 | 0.462 | 0.750 | 0.750 | 0.0099 |
|  |  | LR | 0.724 | 0.769 | 0.688 | 0.813 | 0.0099 |
|  |  | KNN | 0.759 | 0.538 | 0.938 | 0.813 | 0.0099 |
|  | GMN+WMN+FBN | SVM | 0.655 | 0.615 | 0.688 | 0.716 | 0.0099 |
|  |  | RF | 0.621 | 0.538 | 0.688 | 0.750 | 0.0099 |
|  |  | LDA | 0.552 | 0.385 | 0.688 | 0.707 | 0.0099 |
|  |  | LR | 0.586 | 0.462 | 0.688 | 0.707 | 0.0099 |
|  |  | KNN | 0.690 | 0.462 | 0.875 | 0.721 | 0.0099 |

Table 3 the first 5% features ranking of FBN and GMN for SZ-NC groups (.1 represents features from GMN)

| Feature | Weight |
| --- | --- |
| BC_226.1 | 0.044285 |
| BC_78.1 | 0.040193 |
| BC_6 | 0.036937 |
| BC_243.1 | 0.035857 |
| BC_91.1 | 0.03584 |
| BC_201.1 | 0.035401 |
| BC_186 | 0.034675 |
| BC_175 | 0.03453 |
| DC_149.1 | 0.033397 |
| DC_106.1 | 0.033358 |
| NE_196 | 0.032441 |
| BC_86 | 0.030986 |
| BC_164 | 0.030881 |
| DC_94 | 0.030801 |
| DC_78.1 | 0.030743 |
| BC_39 | 0.030684 |
| NE_149.1 | 0.030564 |
| BC_187 | 0.030504 |
| BC_190 | 0.030447 |
| DC_193.1 | 0.030289 |
| DC_22 | 0.029918 |
| DC_77.1 | 0.029744 |
| DC_84.1 | 0.029321 |
| BC_193.1 | 0.028972 |
| DC_36.1 | 0.02894 |
| BC_126.1 | 0.028793 |
| DC_244.1 | 0.028636 |
| NE_122 | 0.028634 |
| NE_84.1 | 0.028482 |
| NE_184.1 | 0.028424 |
| DC_221 | 0.028394 |
| NE_36.1 | 0.028373 |
| BC_38.1 | 0.028372 |
| BC_196 | 0.028281 |
| DC_36 | 0.028096 |
| NE_214 | 0.027929 |
| NE_106.1 | 0.027871 |
| BC_215.1 | 0.02781 |
| DC_147.1 | 0.027798 |
| NE_244.1 | 0.027585 |
| BC_198.1 | 0.027512 |
| DC_81 | 0.027499 |
| BC_199 | 0.027484 |
| BC_155 | 0.027172 |
| NE_193.1 | 0.027147 |
| DC_145 | 0.027072 |
| NE_147.1 | 0.026953 |
| BC_122.1 | 0.026804 |
| BC_184 | 0.026759 |
| DC_164 | 0.026716 |
| BC_139 | 0.026686 |
| BC_160.1 | 0.026206 |
| NE_208.1 | 0.026203 |
| BC_125.1 | 0.026201 |
| NE_54.1 | 0.025986 |
| DC_214 | 0.025912 |
| BC_263 | 0.025881 |
| NE_77.1 | 0.025851 |
| BC_59 | 0.025809 |
| DC_208.1 | 0.025765 |
| DC_123.1 | 0.025703 |
| DC_105.1 | 0.025676 |
| NE_163.1 | 0.025446 |
| BC_159.1 | 0.0254 |
| NE_262.1 | 0.025277 |
| BC_108.1 | 0.025235 |
| DC_184.1 | 0.025204 |
| BC_30 | 0.025189 |
| BC_52.1 | 0.025137 |
| BC_45.1 | 0.025134 |
| BC_204.1 | 0.02508 |
| NE_81 | 0.025078 |
| BC_66 | 0.025021 |
| BC_132 | 0.025009 |
| BC_196.1 | 0.02495 |
| BC_166.1 | 0.024846 |
| BC_212.1 | 0.024756 |
| BC_93 | 0.024623 |
| NE_97.1 | 0.024617 |
| BC_67 | 0.024385 |

Table 4 the first 5% features ranking of GMN and WMN for FESZ-CSZ groups (.1 represents features from WMN)

| Feature | Weight |
| --- | --- |
| DC_130.1 | 0.03103 |
| BC_110 | 0.029897 |
| BC_111.1 | 0.025925 |
| BC_126.1 | 0.025038 |
| DC_204 | 0.025022 |
| BC_81.1 | 0.024611 |
| NE_204 | 0.024153 |
| BC_230.1 | 0.02367 |
| DC_140.1 | 0.023344 |
| DC_223.1 | 0.021669 |
| BC_181 | 0.021226 |
| BC_214.1 | 0.021053 |
| DC_251 | 0.021004 |
| DC_8.1 | 0.020906 |
| DC_77.1 | 0.020893 |
| BC_157 | 0.020835 |
| NE_224 | 0.020599 |
| DC_81.1 | 0.020531 |
| BC_258.1 | 0.020373 |
| BC_203.1 | 0.020216 |
| NE_184 | 0.02008 |
| BC_3.1 | 0.019661 |
| BC_88.1 | 0.019355 |
| DC_123.1 | 0.019294 |
| DC_66.1 | 0.019012 |
| DC_37.1 | 0.018764 |
| BC_55 | 0.018539 |
| BC_263 | 0.018473 |
| NE_208 | 0.018393 |
| DC_268.1 | 0.018321 |
| BC_202 | 0.017976 |
| DC_214.1 | 0.017939 |
| DC_136.1 | 0.017878 |
| DC_208 | 0.017871 |
| BC_73.1 | 0.017824 |
| BC_37.1 | 0.017821 |
| DC_184 | 0.017768 |
| NE_214.1 | 0.017575 |
| NE_129 | 0.01755 |
| NE_162 | 0.017491 |
| DC_184.1 | 0.017448 |
| BC_172.1 | 0.017326 |
| DC_51.1 | 0.01728 |
| DC_242.1 | 0.017268 |
| BC_246.1 | 0.017254 |
| BC_114.1 | 0.017235 |
| BC_79 | 0.017223 |
| BC_176 | 0.017068 |
| BC_170 | 0.017062 |
| BC_91 | 0.016853 |
| DC_162 | 0.016847 |
| DC_110.1 | 0.016839 |
| BC_179.1 | 0.016818 |
| NE_206 | 0.016668 |
| BC_44 | 0.016646 |
| DC_166.1 | 0.016634 |
| BC_46.1 | 0.016618 |
| NE_229 | 0.016559 |
| DC_260.1 | 0.016498 |
| DC_177 | 0.016497 |
| DC_191.1 | 0.016429 |
| BC_171 | 0.016407 |
| NE_230.1 | 0.01635 |
| NE_81.1 | 0.01632 |
| BC_209 | 0.016313 |
| DC_234 | 0.016195 |
| BC_20.1 | 0.016132 |
| NE_139 | 0.016072 |
| BC_208.1 | 0.015972 |
| BC_83 | 0.015899 |
| DC_102 | 0.015769 |
| DC_22.1 | 0.015736 |
| BC_128.1 | 0.015679 |
| DC_206 | 0.015614 |
| BC_67.1 | 0.015545 |
| DC_220.1 | 0.01552 |
| BC_57 | 0.015446 |
| BC_85.1 | 0.015416 |
| BC_149.1 | 0.015413 |
| DC_208.1 | 0.015399 |
